# An analysis of the causes of exhaustion among physicians working in family physician teams during the COVID-19 pandemic in Lithuania

**DOI:** 10.1371/journal.pone.0274360

**Published:** 2022-10-27

**Authors:** Aida Budrevičiūtė, Gediminas Raila, Renata Paukštaitienė, Leonas Valius

**Affiliations:** 1 Independent Scientist, Chief Researcher of the Biomedical Study “Challenges of COVID-19 in Family Medicine”, Vilnius, Lithuania; 2 Department of Family Medicine, Department of Family Medicine, Lithuanian University of Health Sciences, Kaunas, Lithuania; 3 Department of Physics, Mathematics, and Biophysics, Lithuanian University of Health Sciences, Medical Academy, Kaunas, Lithuania; The University of the West Indies, TRINIDAD AND TOBAGO

## Abstract

**Background:**

The COVID-19 pandemic had a severe impact on public life around the world, influencing medicine and health, the economy, employment, science, and education. Health care specialists are key workers who faced extreme challenges posed by the pandemic, including threats to their own lives due to the rapid spread of the virus, a huge increase in workload, and professional burnout syndrome. Analysis of the factors that physicians found most exhausting during the pandemic could lay the groundwork for the effective management of future crises.

**Objective:**

To identify the factors that physicians working in family physician (family and internal medicine) teams found most exhausting during the COVID-19 pandemic in Lithuania and assess their causes.

**Methods:**

An anonymous survey of physicians (*n* = 191) working in family physician teams was carried out from 21 June 2021 to 17 September 2021. Physicians signed an informed consent form prior to completion of the questionnaire. Mixed data analysis was performed, consisting of statistical analysis using the SPSS 27 software and a qualitative causal analysis.

**Results:**

During the pandemic, physicians were most exhausted by: chaotic vaccination priorities (44.5%); unsatisfied patients (52.4%); constantly changing legislation (71.7%); the large workload (75.9%); and the malfunctioning of online systems (81.2%).

**Conclusions:**

Physicians in family physician teams indicated the following aspects that require improvement: service provision; effective work organization for physicians; and the satisfaction of patients with decisions made during the pandemic.

## Introduction

COVID-19 rapidly emerged as a global pandemic, exhibiting mass infection rates and increased mortality rate of infected patients, placing a severe burden on the economy and the health care sector, and necessitating the world-wide mobilization of medical resources [1]. The COVID-19 pandemic forced health care institutions to quickly adapt and plan their activities accordingly, and to reallocate human and material resources [2]. Public and primary health care are key elements of the health care system, and medical staff in both fields had to perform under the extraordinary circumstances brought about by the pandemic to protect the general population [3].

Scientists continue to investigate the COVID-19 pandemic in terms of the spread of the virus and the development of vaccines, assessing clinical aspects (complications, the treatment of symptoms, the progression of chronic comorbidities, and mental health) and measuring the additional expenses incurred in the course of ensuring the effective operation of the health care system during the pandemic. A German study found that the female gender, older age, a higher level of education, and better health care literacy are all factors positively associated with greater adherence to COVID-19 prevention measures [4]. Furthermore, factors such as the male gender, older age, higher education, and adherence to prevention measures increase the likelihood of willingness to undergo vaccination [4]. A study in India found that potential side effects, comorbidities, and unclear information about vaccines were the most common reasons for vaccine resistance within the population [5]. These researchers are also investigating how the dissemination of positive information about the benefits of vaccines could persuade the vaccine-hesitant members of the population to receive vaccination [5].

COVID-19 is caused by the SARS-CoV-2 virus, leading to acute respiratory distress, thrombotic complications, and myocardial injury [6]. Scientific discourse on the treatment of COVID-19 patients suggests that statins, prescribed for lipid reduction, have anti-inflammatory, anti-thrombotic, and immunomodulatory properties and can reduce mortality rates in COVID-19 patients [6]. A study in India found that statin users among COVID-19 patients demonstrated better clinical and laboratory test results compared to non-statin users, therefore supporting the conclusion of the beneficial effects of statins on COVID-19 patients [6].

Remote patient care initiatives (telephone or online video consultations), preventative and control measures, and public information campaigns were introduced to mitigate the effects of the COVID-19 pandemic [7–9]. Recent studies have found that, despite the onset of the pandemic, elderly patients tend to seek in-person consultations with their physicians, forgoing modern technological solutions [10]. Meanwhile, physicians working in primary health care are concerned regarding the abilities of their patients to use technology for service provision [11].

A survey of family physicians conducted in Austria and Germany determined that they were unprepared for the pandemic and lacked sufficient information on how to properly provide health care services to their patients [12]. A survey of primary health care workers (family physicians, nurses, and other medical staff) across several European countries found that the primary health care system had to quickly respond to the following pandemic-induced challenges: restricted physician–patient contact; remote consultations; increased workload for medical staff; professional burnout; and conflicting and often deficient information about managing the pandemic [13].

The key question posed by this study is: What are the reasons behind the exhaustion experienced by physicians in family physician teams during the COVID-19 pandemic?

## Methods

### Study population and criteria for sample selection

In this study we conducted the survey of family and internal medicine physicians, not evaluating the opinion of juniors/interns physicians (students of medicine). Our decision was based on the reason that the most important role in COVID-19 pandemic management and final decision makers were only family and internal medicine physicians, when interns plays just supporting function in primary health care level during the COVID-19 pandemic. Sample size was representative of the age, gender, and distribution of physicians in different counties in Lithuania. The 50/50 principle was applied when selecting respondents to ensure the participation of physicians from both public and private primary health care institutions (PHCIs). According to the data provided by the Institute of Hygiene, 1,903 family physicians and 238 internal medicine physicians were employed by PHCIs and care homes at the end of 2020 (Table 1).

**Table 1:** The distribution of family and internal medicine physicians in Lithuanian counties.

County	Number of family physicians, total	Number of internal medicine physicians, total	Number of family and internal medicine physicians in the county (*N*), total	Relative distribution of physicians in counties, % of *N*	Estimated number of physicians from each county to be included in the survey
Vilnius	596	87	683	32	127
Kaunas	452	40	492	23	91
Klaipėda	203	14	217	10	40
Šiauliai	167	14	181	8	34
Panevėžys	118	23	141	7	26
Utena	79	13	92	4	17
Marijampolė	84	14	98	5	18
Tauragė	48	7	55	3	10
Telšiai	80	9	89	4	17
Alytus	76	17	93	4	17
**Total**	**1903**	**238**	**2141**	**100**	**398**

Source: The Institute of Hygiene, end of 2020 data.

The sample size of 385 physicians was calculated using the Paniotto formula (confidence level 95%; margin of error 0.05), and the researchers intended to survey 398 respondents (19% of the total population). Using the data provided by the State Health Care Accreditation Agency under the Ministry of Health, the researchers determined a representative number of respondents according to age and gender (Table 2).

**Table 2:** The distribution of family and internal medicine physicians in the study population based on age and gender.

Distribution based on age and gender		Number of internal medicine physicians, total	Number of family physicians, total	Total (*N*)	Relative distribution based on age and gender, % of total study population *N*	Percentage of age group, %	Estimated number of physicians to be included in the survey (N = 398)
Under 40	Male	31	88	119	13	25	13
	Female	137	671	808	87		87
**Total**		**168**	**759**	**927**	**100**		**100**
41–50	Male	7	62	69	17	10	7
	Female	39	297	336	83		32
**Total**		**46**	**359**	**405**	**100**		**39**
51–60	Male	64	83	147	16	25	16
	Female	332	460	792	84		84
**Total**		**396**	**543**	**939**	**100**		**100**
61–70	Male	105	76	181	15	33	20
	Female	376	678	1054	85		111
**Total**		**481**	**754**	**1235**	**100**		**131**
71+	Male	28	24	52	18	7	5
	Female	130	101	231	82		23
**Total**		**158**	**125**	**283**	**100**	**100**	**28**

Source: State Health Care Accreditation Agency under the Ministry of Health, 2021 data.

### The characteristics of the sample size

**Chart 1:**
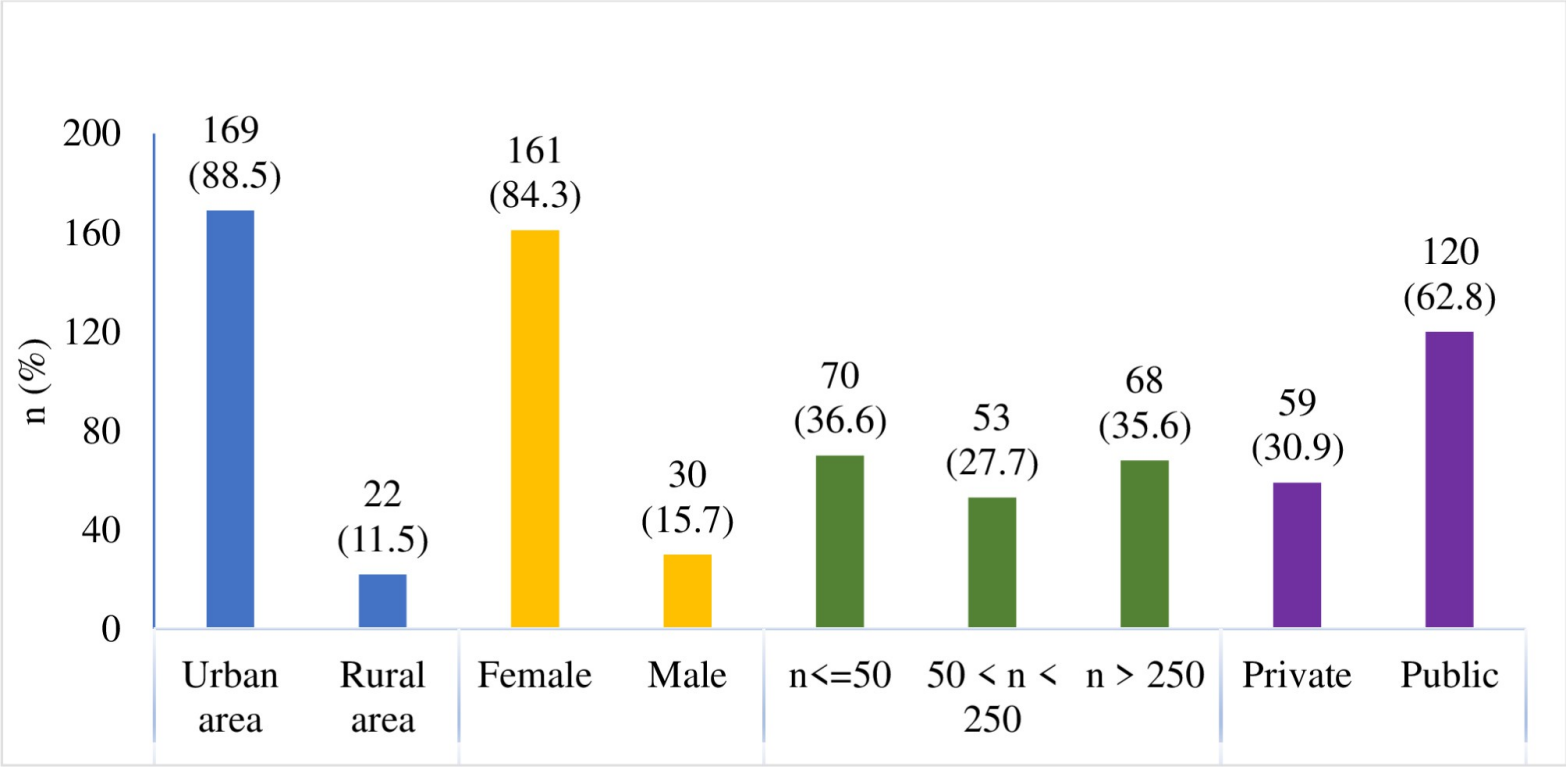
The characteristics of the study population.

A quantitative study was conducted from 21 June 2021 to 17 September 2021, and a total of 398 questionnaires were sent out. Of these, 191 completed questionnaires were used for analysis and 4 were invalid (incomplete), resulting in a response rate of 48%. Respondents didn’t remark the reason of answering not all questions of the questionnaire and the scientists make just assumptions as unwillingness to fulfill all questionnaire, lack of time, etc. The questionnaires were completed by 9% of the total population. Of the 39 PHCIs randomly selected for the study, 11 were public and 28 were private. The respondents were distributed as follows: 31% were employed by a private PHCI; 63% were employed by a public PHCI; and 6% were employed by both public and private PHCIs. In terms of location, 88% of respondents worked in urban areas and 12% worked in rural areas. According to gender, 84.3% of respondents were female and 15.7% were male. The distribution of respondents based on the size of their place of work was as follows: employees of very small PHCIs (up to 10 employees) made up 9% of the sample; employees of small PHCIs (11–50 employees)– 28%; employees of medium-sized PCHIs (51–250 employees)– 28%; and employees of large PHCIs (over 250 employees)– 35%. The youngest respondent–physician was 24 years old, the oldest was 85, and the average age of the respondents was 53.14 (standard deviation *s* = 14.68, median 56). The average work experience of the respondents was 25.88 years (*s* = 15.68, median 30), the shortest work experience was 1 year, and the longest was 55 years. There was a statistically significant difference in the duration of employment at the current workplace based on the size of the health care institution (*H* = 6.88, *p* = 0.032). Pairwise comparisons of the Kruskal–Wallis criterion demonstrated a statistically significant difference between the duration of employment of staff at large (5.25–38 years, median 22.50, *p* = 0.033) and small (3–20 years, median 13, *p* = 0.032) health care institutions. A statistically significant difference was observed between the duration of employment of respondents who worked in a public PHCI (5–38 years, median 21.5) and a private PHCI (2–18 years, median 8; *Z* = 4.21; *p* < 0.001). A total of 161 women (84.3%) and 30 men (15.7%) participated in the study (Chart 1).

### Statistical analysis

In response to the question of what they found most exhausting during the COVID-19 pandemic, the respondents had to assess each factor from 1 (*not exhausting*) to 5 (*very exhausting*). The results of the survey demonstrated that the respondents had highly inconsistent views towards which factors they found exhausting: some factors were found very exhausting by nearly all respondents, whereas other factors had limited impact. Therefore, the division of the respondents into groups based on the score they assigned to each factor created a variance in the number of groups. IBM SPSS Statistics 27 was used for data analysis. The independence (compatibility) criterion χ^2^ (with Yates correction for binary data) was used for qualitative data analysis. The results were described in terms of frequency and relative frequency (percentage), and the statistics of the criterion χ^2^. Quantitative variables did not meet the conditions of normal distribution and were therefore analyzed using nonparametric criteria. The Mann–Whitney test was used to compare differences between two groups, and the Kruskal–Wallis test with multiple pairwise comparisons was used when comparing more than two groups. The results were presented as medians and quartiles of the values of lower (Q_0.25_) and upper (Q_0.75_) quantitative variables and the statistics of the applied criterion (Mann–Whitney–*Z*; Kruskal–Wallis–*H*). The observed differences were considered statistically significant if the calculated *p*-value was lower than the level of significance (α = 0.05).

### Ethical approval

Permission (No. BE-2-63) to conduct this research was issued on 15 June 2021 by the Kaunas Regional Committee of Biomedical Research Ethics. The written informed consent forms were obtained from respondents prior to completion of the questionnaire.

## Results

**Chart 2:**
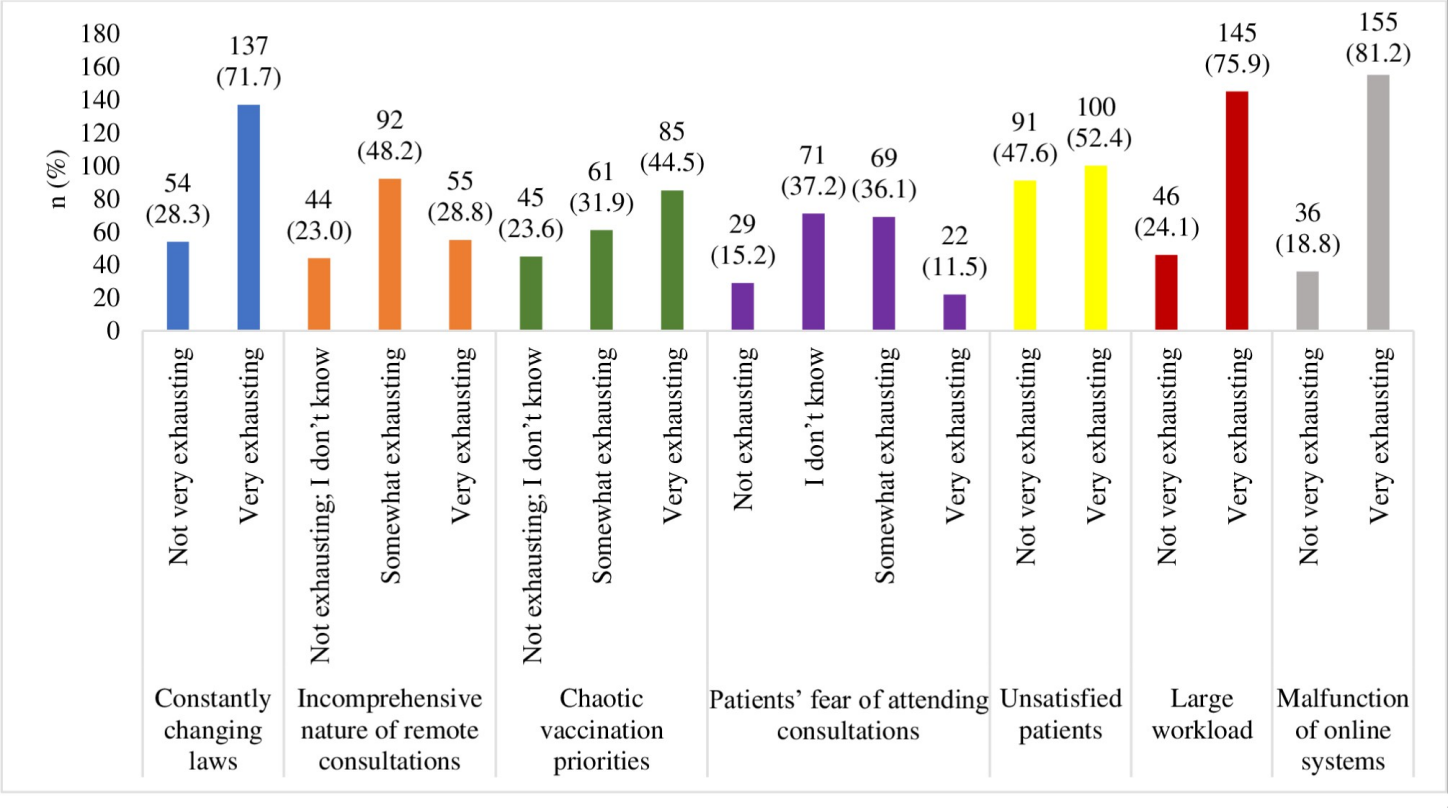
The distribution of respondents according to the groups of factors they found exhausting.

During the pandemic, physicians working in family physician teams were most exhausted (on a scale of 1 to 5) by: constantly changing legislation (71.7%); chaotic vaccination priorities (44.5%); unsatisfied patients (52.4%); the large workload (75.9%); and the malfunctioning of online systems (81.2%) (Chart 2).

The incomprehensive nature of consultations was “somewhat exhausting” (indicated by a score of less than 5) to 48.2% of the respondents, whilst patients’ fears of attending in-person consultations were “somewhat exhausting” to 36.1% of the respondents. The distribution of respondents was not homogeneous among PHCIs of different sizes regarding this factor (χ^2^ = 9.974; *p* = 0.041). Pairwise comparisons demonstrated that statistically significantly more respondents from small PHCIs claimed the issue to be “somewhat exhausting” than “very exhausting” (42 (45.7%) and 14 (25.5%), respectively, *p* < 0.05). In medium-sized PHCIs, respondents who indicated the issue as “very exhausting” were more frequent than those who found it “somewhat exhausting” (20 (36.4%) and 17 (18.5%), respectively, *p* < 0.05). The data showed that the difference between the level of exhaustion expressed by males and females regarding chaotic vaccination priorities was statistically significant (χ^2^ = 6.69; *p* = 0.035). Pairwise comparisons demonstrated that more female respondents indicated being “somewhat exhausted” by chaotic vaccination priorities than “not exhausted” (56 (91.8%) and 33 (73.3%), respectively, *p* < 0.05). More male respondents were “not exhausted” by the chaotic vaccination priorities than found it “somewhat exhausting” (12 (26.7%) and 5 (8.2%), respectively, *p* < 0.05). Respondents from urban and rural areas experienced diverging levels of exhaustion related to patients’ fears of attending in-person consultations (χ^2^ = 9.45; p = 0.009). All respondents who found this issue “very exhausting” worked in a city (22 (100%)), whereas rural-based respondents more frequently found it “somewhat exhausting” (14 (20.3%)). The age difference of the respondents was statistically significant (*H* = 16.34, *p* = 0.001): respondents who found this issue “somewhat exhausting” were statistically significantly older (median 62 (52.5–68)) than those who were “unsure” (53 (35–63), *p* = 0.02) or “not exhausted” (47 (37–58), *p* = 0.002). Exhaustion due to increased workload was statistically significantly dependent on the size of the PHCI (χ^2^ = 9.89; *p* = 0.007). A higher proportion of the respondents who found the extra workload “very exhausting” worked in medium-sized PHCIs than those who found it “not very exhausting” (47 (32.4%) and 6 (13%), respectively, *p* < 0.05). A higher proportion of the respondents who did not find the increased workload exhausting worked in large PHCIs than those who found it “very exhausting” (24 (52.2%) and 44 (30.3%); *p* < 0.05).

The qualitative analysis of causality demonstrated that physicians working in family physician teams were exhausted by bureaucracy and their work organization during the COVID-19 pandemic (Table 3).

**Table 3:** The qualitative analysis of the reasons for exhaustion among physicians during the COVID-19 pandemic.

Size of PHCI based on number of staff	Categories of reasons	Reasons for exhaustion among physicians during the COVID-19 pandemic
Large PHCIs (>250)	Patient registration	Patients calling on the phone at their convenience, without making an appointment in advance (female, 72, public PHCI).
	Workload	Patients who contracted the COVID-19 virus increased the existing workload and impeded planned patient care (female, 70, public PHCI).
	Work organization	The role of a family doctor during a quarantine has not been defined and approved for the public (female, 57, public PHCI).
	Bureaucracy	Additional tasks that do not necessarily require a medical degree (female, 50, public PHCI).
		The burden of bureaucracy (female, 32, public PHCI).
		Additional obligations in the form of social work assigned to family physicians by order of the Ministry of Health that are not related to treatment or preventative health care (female, 60, public PHCI).
Medium-sized PHCIs (50–250)	Inter-institutional collaboration	Public health bureaus aren’t involved enough in the vaccination process (information; teaching people why vaccination is necessary; prevention of infectious disease; etc.) (female, 37, public PHCI).
	Patient-doctor contact	Disrespectful patients (female, 37, public PHCI).
	Timely disease diagnostics	Patients who were too afraid to consult a doctor at the start of the pandemic and left their conditions untreated (female, 35, public PHCI).
	Work organization	Very little time remains for normal interaction with a patient (female, 69, public PHCI).
	Bureaucracy	Non-medical duties (issuing certificates) (female, 49, private PHCI).
	Collaboration between family physicians and other specialists	The procedure for accessing specialist care should be easier; the social problems of patients and epidemiological issues that do not require treatment should be delegated to the relevant agencies (female, 31, public PHCI).
	Management of information	Lack of credible and clear information (female, 58, private PHCI).
Very small and small PHCIs (< = 50)	Work organization	Scheduling in-person and telephone consultations (female, 42, public PHCI).
		High volume of telephone consultations, sluggish online system, and unlimited consultations throughout the day (female, 50, private PHCI).
		The processing of disability and capacity for work assessment forms, and the huge volume of work (female, 50, private PHCI).
	Job functions of a doctor	Functions delegated to a family doctor that have nothing to do with treatment: issuing COVID-19 “passports”; performing PCR tests for asymptomatic patients; issuing various certificates (female, 62, private and public PHCI).
	Bureaucracy	Introduction of new bureaucratic procedures (male, 29, private PHCI).
	Vaccination benefits	Anti-vaxxers who are generally intolerant of other opinions (female, 40, private PHCI)
	Patient-doctor contact	People’s anger, irritation, and impatience (female, 65, public PHCI).

## Discussion

In Lithuanian primary health care, support staff, such as receptionists, nurses, assistants, and medical students, participated in the management of the COVID-19 pandemic. Receptionists managed patient flows, providing remote check-in services for appointments with physicians or fever clinics. Nurses and their assistants performed vaccination procedures for patients. Volunteer medical students provided consultations (for example, by recording patient problems, symptoms, and anamneses) by phone, relaying patients to fever clinics or family physicians. The COVID-19 pandemic saw an increase in the number of undiagnosed new COVID-19 cases, facilitated by patients’ fears of attending in-person consultations with a physician. It is important to take into account the opinions expressed by physicians in this study and to effectively manage patient flows (acute cases of the virus, cases of new diseases, and cases of previously diagnosed chronic diseases) in the event of a future pandemic in Lithuania.

One study in Poland revealed that the most common reasons behind a patient’s refusal to vaccinate were: concerns about side effects; mistrust in the vaccine; and the belief that COVID-19 will not affect them [14]. Meanwhile, a study in Germany found that health care workers were most concerned about the following long- and short-term side effects of the vaccine: reactions; allergic reactions; impact on daily life; immune response; neurological side effects; and currently unknown long-term consequences on health [15]. Our study results show that family and internal medicine physicians were most exhausted by chaotic vaccination priorities and unsatisfied patients during the COVID-19 pandemic. Improving physician–patient contact (increasing patients’ confidence in vaccines through the dissemination of reliable information and positive communication with the public) is critical in such uncertain circumstances. Increased satisfaction among physicians and patients is conditional upon a national health strategy for crisis management that encompasses effective collaboration between institutions, family physicians, and other specialists on the one hand, and physicians and patients on the other.

Researchers are investigating the crisis in primary health care caused by the pandemic in the following areas: the impact of the COVID-19 pandemic on primary health care service provision (reduced accessibility and quality of services, decreased care for non-COVID-19 patients); the impact of COVID-19 on the health of patients (care for patients with chronic diseases, mental health services); and the use of telemedicine in primary health care provision to help improve access and quality of service [10]. A study in Canada found that medical staff felt exhausted by their new professional responsibilities, increased workload, and the demand for innovation [16]. An analysis of appointments with primary health care specialists exposed an increase in anxiety- and depression-related visits, while consultations for preventative and chronic non-infectious disease management decreased [17]. During the pandemic, health care specialists expressed concern over: their own physical health; workload; ethical, moral, and professional challenges; collaboration with families and colleagues; communication with other institutions and the public; and access to knowledge and information [18]. An online survey of medical staff in Singapore indicated the occurrence of stress, anxiety, and professional burnout syndrome during the COVID-19 pandemic [19]. A study in Estonia found that 52.2% of the population experienced excessive stress due to the pandemic, with a higher prevalence found among: females; younger age groups; respondents with better self-perception of infection risk; respondents exhibiting symptoms of respiratory diseases; and groups with worse self-rated health [20]. A study in Poland demonstrated that care nurses frequently experience tremendous psychological pressure due to increased workload in a high-risk environment [21]. In our study, we found that family and internal medicine physicians experienced constantly changing legislation (71.7%) and a large workload (75.9%) during the COVID-19 pandemic. Clearly defined principles relating to the job functions of physicians and the organization of their work during the pandemic would assist in avoiding diagnostic errors and would lower the level of bureaucracy.

The widespread introduction of remote consultations during lockdown increased the number of phone calls from patients to physicians, and posed certain technological challenges owing to the lack of a national strategy for managing the pandemic [22]. A study in Latvia determined that the number of in-person consultations provided by family and specialist physicians for patients with non-communicable diseases decreased during the COVID-19 pandemic, instead being substituted with remote consultations, and thus growing the demand for technological solutions for telemedicine [23]. Meanwhile, our study found that a staggering 81.2% of family and internal medicine physicians encountered technological issues during remote consultations. Researchers observed certain key areas to consider in preparation for future crises in health care, noting that the primary health care system must: quickly respond to increased health problems in patients during the pandemic and properly manage patient flow; improve access to and services provided by physicians; and provide managed, reliable information to the public [24].

The key strength of this study is in the quality of the respondents, who consisted of physicians in family physician teams responsible for promoting a patient’s health. The mixed (statistical and qualitative) analysis of the data provided valuable insights into the factors and causes of exhaustion among physicians during the COVID-19 pandemic. However, this study did not involve any pre-pandemic data. The results of this study demonstrate the opinions of Lithuanian family and internal medicine physicians regarding the factors that they found exhausting during the pandemic, but the same factors cannot be considered typical of the wider European context. Future researchers could make some general conclusions if this study was replicated in other countries.

## Conclusion

During the pandemic, physicians were most exhausted by constantly changing legislation, chaotic vaccination priorities, unsatisfied patients, the large workload, and the malfunctioning of online systems. The large workload was a significant cause of exhaustion for respondents working in medium-sized PCHIs. Physicians also experienced exhaustion caused by increased bureaucracy and the unclear and poorly defined organization of their work.

## References

[1] Umakanthan S, Sahu P, Ranade AV, Bukelo MM, Rao JS, Abrahao-Machado LF, et al. Origin, transmission, diagnosis and management of coronavirus disease 2019 (COVID-19). Postgrad Med J. 2020 Dec;96(1142):753–758. doi: 10.1136/postgradmedj-2020-138234 32563999 PMC10016932

[2] COVID-19: occupational health and safety for health workers, interim guidance, 2 February 2021. World Health Organization, access on 5th of August 2021 in site https://www.who.int/publications/i/item/WHO-2019-nCoV-HCW_advice-2021.1.

[3] Mash B. Addendum: Primary care management of the coronavirus (COVID-19). South African Family Practice, 2020;62(1):a5115. doi: 10.4102/safp.v62i1.5115 32634001 PMC7577340

[4] Umakanthan S, Lawrence S. Predictors of COVID-19 vaccine hesitancy in Germany: a cross-sectional, population-based study Postgraduate Medical Journal, Published Online First: 03 February 2022. doi: 10.1136/postgradmedj-2021-141365 37062994

[5] Umakanthan S, Patil S, Subramaniam N, Sharma R. COVID-19 vaccine hesitancy and resistance in India explored through a population-based longitudinal survey. Vaccines, 2021,9,1064. doi: 10.3390/vaccines9101064 34696172 PMC8537475

[6] Umakanthan S, Senthil S, John S, Madhavan MK, Das J, Patil S, et al. The effect of statins on clinical outcome among hospitalized patients with COVID-19: a multi-centric cohort study. Front. Pharmacol. 2022;13:742273. doi: 10.3389/fphar.2022.742273 35865966 PMC9294274

[7] Neves AL, Li E, Gupta PP, Fontana G, Darzi A. Virtual primary care in high-income countries during the COVID-19 pandemic: policy responses and lessons for the future. European Journal of General Practice, 2021;27(1):241–247. doi: 10.1080/13814788.2021.1965120 34431426 PMC8404680

[8] Solans O et al. Characteristics of citizens and their use of teleconsultations in primary care in the Catalan Public Health System before and during the COVID-19 pandemic: restrospective descriptive cross-sectional study. Journal of Medical Internet Research, 2021;23(5):e28629. doi: 10.2196/28629 33970867 PMC8163495

[9] Coronavirus COVID-19 Global cases by the center for systems science and engineering (CSSE) at Johns Hopkins, retrieved 5th of August 2021, access in site https://coronavirus.jhu.edu/map.html.

[10] Lim J et al. COVID-19’s impact on primary care and related mitigation strategies: a scoping review. European Journal of General Practice, 2021;27(1):166–175. doi: 10.1080/13814788.2021.1946681 34282695 PMC8293960

[11] Mohammed HT, Hyseni L, Bui V, Gerritsen B, Fuller K, Sung J, et al. Exploring the use and challenges of implementing virtual visits during COVID-19 in primary care and lessons for sustained use. PloS One, 2021;16(6):e0253665. doi: 10.1371/journal.pone.0253665 34166441 PMC8224904

[12] Siebenhofer A. et al. COVI-Prim survey: challenges for Austrian and German general practitioners during initial phase of COVID-19. PloS One, 2021;16(6):e0251736. doi: 10.1371/journal.pone.0251736 34111120 PMC8191874

[13] Wanat M. et al. Transformation of primary care during the COVID-19 pandemic: experiences of healthcare professionals in eight European countries. British Journal of General Practice, 2021;71(709):e634–e642. doi: 10.3399/BJGP.2020.1112 33979303 PMC8274627

[14] Zarębska-Michaluk D, Rzymski P, Moniuszko-Malinowska A, Brzdęk M, Martonik D, Rorat M, et al. Does hospitalization change the perception of COVID-19 vaccines among unvaccinated patients? Vaccines, 2022;10(3):476. doi: 10.3390/vaccines10030476 35335108 PMC8950102

[15] Holzmann-Littig C, Frank T, Schmaderer C, Braunisch MC, Renders L, Kranke P, et al. COVID-19 vaccines: fear of side effects among German Health Care Workers. Vaccines, 2022,10,689. doi: 10.3390/vaccines10050689 35632445 PMC9146316

[16] Ashcroft R et al. Primary care teams‘ experiences of delivering mental health care during the COVID-19 pandemic: a qualitative study. BMC Family Practice, 2021;22:143. doi: 10.1186/s12875-021-01496-8 34210284 PMC8248293

[17] Stephenson E, Butt DA, Gronsbell J, Ji C, O‘Neill B, Crampton N, et al. Changes in the top 25 reasons for primary care visits during the COVID-19 pandemic in a high–COVID region of Canada. PloS One, 2021;16(8):e0255992. doi: 10.1371/journal.pone.0255992 34383844 PMC8360367

[18] Billings J, Ching BChF, Gkofa V, Griene T, Bloomfield M. Experiences of frontline healthcare workers and their views about support during COVID-19 and previous pandemics: a systematic review and qualitative meta-synthesis. BMC Health Services Research, 2021;21:923. doi: 10.1186/s12913-021-06917-z 34488733 PMC8419805

[19] Teo I et al. Healthcare worker stress, anxiety and burnout during the COVID-19 pandemic in Singapore: A 6-month multi-centre prospective study. PloS One, 2021;16(10):e0258866. doi: 10.1371/journal.pone.0258866 34679110 PMC8535445

[20] Reile R, Kullamaa L, Hallik R, Innos K, Kukk M, Laidra K, et al. Perceived stress during the first wave of COVID-19 outbreak: results from nationwide cross-sectional study in Estonia. Front. Public Health, 2021;9:564706. doi: 10.3389/fpubh.2021.564706 34222158 PMC8249769

[21] Tomaszewska K, Majchrowicz B, Delong M. Impact of SARS-CoV-2 pandemic on psychosocial burden and job satisfaction of long-term care nurses in Poland. Int. J. Environ. Res. Public Health, 2022,19,3555. doi: 10.3390/ijerph19063555 35329241 PMC8953701

[22] DeVoe JE, Bazemore A. Primary care in the COVID-19 pandemic: essential, and inspiring. The Journal of the American Board of Family Medicine, 2021;34. doi: 10.3122/jabfm.2021.S1.200631 33622807

[23] Kursīte M, Stars I, Strēle I, Gobiņa I, Ķīvīte-Urtāne A, Behmane D, et al. A mixed-method study on the provision of remote consultations for non-communicable disease patients during the first wave of the COVID-19 pandemic in Latvia: lessons for the future. BMC Health Services Research, 2022;22:263. doi: 10.1186/s12913-022-07634-x 35219328 PMC8881750

[24] Rawaf S, Allen LN, Stigler FL, Kringos D, Yamamoto Q, Weel van Ch. On behalf the Global Forum on Universal Health Coverage and Primary Health Care. Lessons on the COVID-19 pandemic, for and by primary health care professionals worldwide. European Journal of General Practice, 2020;26(1):129–133. doi: 10.1080/13814788.2020.1820479 32985278 PMC7534357

